# Validity and Reliability of a Water Frequency Questionnaire to Estimate Daily Total Water Intake in Adults

**DOI:** 10.3389/fnut.2021.676697

**Published:** 2021-06-14

**Authors:** Abigail T. Colburn, Evan C. Johnson, François Péronnet, Lisa T. Jansen, Catalina Capitan-Jimenez, J. D. Adams, Isabelle Guelinckx, Erica T. Perrier, Andy Mauromoustakos, Stavros A. Kavouras

**Affiliations:** ^1^Hydration Science Lab, College of Health Solutions, Arizona State University, Phoenix, AZ, United States; ^2^Human Integrated Physiology Laboratory, University of Wyoming, Laramie, WY, United States; ^3^Department of Kinesiology, University of Montreal, Montreal, QC, Canada; ^4^Division of Endocrinology, New Balance Foundation Obesity Prevention Center, Boston Children's Hospital, Boston, MA, United States; ^5^Harvard Medical School, Boston, MA, United States; ^6^Department of Nutrition, Universidad Hispanoamericana, San Jose, Costa Rica; ^7^Department Health and Human Performance, College of Charleston, Charleston, SC, United States; ^8^Health, Hydration and Nutrition Science, Danone Research, Palaisaeu, France; ^9^Agricultural Statistics Laboratory, University of Arkansas, Fayetteville, AR, United States

**Keywords:** dietary assessment, hydration, nutrition methodology, self-report, underhydration, water

## Abstract

The purpose of this investigation was to assess the validity and reliability of a seven-day water frequency questionnaire (TWI-FQ) to estimate daily total water intake (TWI) in comparison to a water turnover objective reference value via deuterium oxide (D_2_O). Data collection occurred over 3 weeks, with a wash-out period during week two. Healthy adults (*n* = 98; 52% female; 41 ± 14 y; BMI, 26.4 ± 5.5 kg·m^−2^) retrospectively self-reported consumption frequencies of 17 liquids and 35 foods with specified volumes/amounts for weeks one and three via TWI-FQ. Standard water content values were utilized to determine the volume of water consumed from each liquid and food for calculation of mean daily TWI for each week. Diet records were completed daily during week two to estimate metabolic water production. To assess validity of the TWI-FQ, participants consumed D_2_O at the start of each week and provided urine samples immediately before ingestion, the following day, and at the end of the week to calculate water turnover. Metabolic water was subtracted from water turnover to estimate TWI. TWI-FQ validity was assessed via Bland-Altman plot for multiple observations. Reliability was assessed via intraclass correlation and Pearson's correlation between weeks. TWI-FQ significantly underestimated D_2_O TWI by −350 ± 1,431 mL·d^−1^ (95% confidence interval (CI): −551, −149 mL·d^−1^). TWI-FQ TWI was significantly correlated (*r* = 0.707, *P* <0.01) and not different (198 ± 1,180 mL·d^−1^, 95% CI: −38, 435 mL·d^−1^) between weeks. TWI-FQ intraclass correlation = 0.706 was significant [95% CI: 0.591, 0.793; *F*_(97, 98)_ = 5.799], indicating moderate test-retest reliability. While this tool would not be suitable for individual TWI assessment, the magnitude of bias may be acceptable for assessment at the sample-level.

## Introduction

A limited ability to accurately assess water intake at a population-level has likely slowed progress in elucidating the impact of water intake on health. Some evidence suggests that low water consumption and underhydration are associated with adverse health outcomes including chronic kidney disease and diabetes (1–3). Similarly, the Institute of Medicine suggests dehydration may be related to numerous detrimental health outcomes including cardiovascular strain, urinary tract infection, and diabetes dysregulation (4). Conversely, increased water intake is associated with positive health outcomes including reduced risk for kidney stones (4) and urinary tract infections (5) as well as augmented glucose regulation (6) and adolescent cognitive performance (7). Thus, there appears to be an inverse relationship between water intake and health risk. However, evidence supporting these associations is not sufficient to establish total water intake (TWI) recommendations beyond an Adequate Intake, the least specific recommendation included in the Institute of Medicine's dietary reference intakes (4). Due to the wide range of TWI volumes that allowed individuals to maintain adequate serum osmolality, the Adequate Intake is the median value of the TWI volumes observed in the Third National Health and Nutrition Examination Survey (4). These high variations have largely been attributed to differences in culture, climate, and/or physical activity (4). However, measurement error in TWI assessment and lack of a standard assessment tool could exacerbate these variations.

The TWI Adequate Intake recommendations encompass water consumed from all foods (~20% TWI) and liquids (~80% TWI) (4). The current recommendation is based upon the National Health and Nutrition Examination Survey 24-h diet recalls, which were conducted before utilization of the United States Department of Agriculture's Automated Multiple-Pass Method, which is a validated method for energy and nutrient intake, but not TWI (8). Additionally, trained interviewers prompt participants to choose an occasion for every food item on the record, most of which are meals (9). However, beverage intake occurs more continuously throughout the day. Individuals have 0 – 19 drinking occasions per day (of water only), with an average time of 3 h between drinking occasions (range 1–17 h) (10). This has been observed when TWI was compared between a fluid-specific tool and the United Kingdom's National Diet and Nutrition Survey, which utilizes food diaries (11). The fluid-specific tool revealed that 70% of beverage consumption occurred outside of meals. In Indonesian populations, TWI estimated from a 7-day fluid diary was significantly greater than that from the 24-h dietary recall, by 382 mL (12). Additionally, the 24-h dietary recall captured 2.2 fewer drinking occasions (6.7 vs. 8.9 occasions). Consequently, current dietary assessments which have not been validated for water intake are not necessarily suitable for drinking behavior.

To date, investigators have not been able to identify a method to estimate TWI that is comparable to an objective reference value, such as that obtained from water turnover by dilution of deuterium oxide (D_2_O) (13) corrected for metabolic water, which is costly and impractical for population-level use. Recently, our group and others have begun to advance the field through development and validation of fluid-specific assessment tools (14–19). Compared to 24-h recalls, which are subject to bias from day-to-day variation in consumption, frequency questionnaires are more likely to capture usual intake (20). However, only relative validation, via dual reporting, has been assessed for prior beverage frequency questionnaires with comparison of water intake estimates against self-reported 24-h records (14–17). As the 24-h diet record and new questionnaires under assessment for validation are both self-reporting instruments, sources of error will overlap between the instruments and be correlated. Additionally, validation through dual recording will not distinguish inaccuracies if they are reported on both assessments. Dual recording could also deceptively improve accuracy of the new questionnaire, and therefore falsely show validation, as recording intake in diet records in days leading up to a frequency recall will likely improve recall accuracy.

We recently utilized D_2_O to validate Liq.In^7^, a 7-day fluid diary, to record all beverage intake over seven-days. While it has been shown to be an accurate recording instrument for TWI volume, the seven-days of recording impose substantial subject burden (18). Additionally, Liq.In^7^ only captures water from liquids, and not TWI. However, there is limited evidence from the US and Europe supporting the current belief that TWI is ~80% water from liquids. In fact, those with high and low TWI have been observed to consume a similar amount of water from food (~0.6 L·d^−1^), resulting in substantially different contributions to TWI. For instance, water from food comprised ~23% of TWI in those with high TWI, while ~47% in those with low TWI (21). Consequently, liquid-only assessments may be preferable in studies where precise recording of fluid intake is important but may elucidate misleading results in terms of TWI. To address this gap, we developed a total water intake frequency questionnaire (TWI-FQ) that prompts individuals to recall water intake from food and beverages over a 7-day period. The purpose of this investigation was to assess the reliability and validity of the TWI-FQ to estimate TWI as compared to the value obtained with dilution of D_2_O, corrected for metabolic water.

## Materials and Methods

### Subjects

Potential healthy participants (*n* = 262, 18 – 65 y) were recruited from Northwest Arkansas, and provided informed consent acknowledging the risks and benefits of participating in the study (Supplementary Figure 1). Following completion of a medical history questionnaire, individuals were excluded if they satisfied any of the following criteria: (1) unable to understand and write English, (2) currently pregnant or breastfeeding, (3) previous surgical operation on digestive tract (excluding appendectomy), (4) drug treatment within 15-days prior to the start of the study, (5) exercise > 4 h·week^−1^, (6) dietary changes within the last month, or (7) changes in body weight > 2.5 kg within the last month. Volunteers with clinically relevant diseases that could alter fluid balance (i.e., relevant metabolic, cardiovascular, hematologic, hepatic, gastrointestinal, renal, pulmonary, endocrine or psychiatric history of disease) were not enrolled.

Ultimately, 103 individuals received medical clearance, met all criteria, consented to voluntary participation, enrolled, and completed the study protocol. Data from five participants were excluded due to missing data that prevented calculation of TWI through TWI-FQ or dilution of D_2_O during weeks one or three. Participant demographics are presented in Table 1. Data collection occurred May – December 2014 in Fayetteville, Arkansas, USA (ambient temperature, 17.2 ± 8.4°C). This protocol was approved by the University's institutional review board and biosafety committee (protocol no. 14-03-555) and was conducted in compliance with the Helsinki Declaration as revised in 1983.

**Table 1:** Baseline sample demographics by sex and age group.

	**Women**			**Men**			**All participants**
**Age range, *y***	**18–29**	**30–49**	**50–65**	**18–29**	**30–49**	**50–65**	**41 ± 14**
Participants, *n*	14	22	15	12	21	14	98
Height^a^, *m*	1.66 ± 0.06	1.62 ± 0.06	1.64 ± 0.08	1.75 ± 0.04	1.75 ± 0.07	1.81 ± 0.07	1.70 ± 0.09
Weight^a^, kg	68.9 ± 19.9	75.4 ± 17.6	69.6 ± 11.7	74.1 ± 18.1	79.2 ± 13.3	89.0 ± 14.2	76.2 ± 16.8
BMI^a^, kg·m^−2^	25.0 ± 6.8	28.8 ± 6.7	25.8 ± 4.0	24.5 ± 6.5	25.7 ± 4.0	27.1 ± 3.7	26.4 ± 5.5
Total Body Water^a^^,^ ^b^, L	34.5 ± 6.9	33.1 ± 4.7	30.7 ± 3.3	39.6 ± 5.2	44.3 ± 6.4	46.2 ± 6.0	38.0 ± 8.0
Total Body Water^a^^,^ ^b^, %BM	51.1 ± 6.0	45.0 ± 7.0	44.6 ± 4.0	54.8 ± 7.6	56.4 ± 5.1	52.4 ± 4.8	50.5 ± 7.4

*BMI, body mass index; %BM, total body water as a percentage of body mass*.

a *Values are presented as mean ± standard deviation*.

b *Total body water is the average of values computed at weeks 1 and 3*.

### Questionnaire Development

The TWI-FQ is a 59-item water intake assessment that quantitatively assesses frequency and volume of TWI within the period of a week. The first and second page of the questionnaire consisted of 24 and 35 items to assess water from liquid and food, respectively. The TWI-FQ included 17 liquid types with specified volumes (e.g., water [8 fl oz cup]). Water was further broken into eight occasions of consumption to include periods that may be forgotten in traditional meal- and snack-focused questionnaires (e.g., before breakfast, between lunch & dinner, during your sleep). Nine frequency options were included, ranging from “Never or <1 per week” to “7+ per day.” The TWI-FQ also includes four overarching food categories (vegetables; fruits; cheese, egg, meats; & bread, cereal, starches). Within categories, food types were listed with specified quantities (e.g., mango, pineapple [1 cup], pizza [1 slice]). Eight frequency options were included, ranging from “Never or <1 per week” to “6+ per day.” The TWI-FQ has a Flesch-Kincaid grade level of 8.4 and a completion time of ~5 min.

The TWI-FQ is visually similar to the validated Harvard Willett Food Frequency Questionnaire (Harvard T.H. Chan School of Public Health, Department of Nutrition) (22). While this questionnaire includes a section on beverage intake, reproducibility and validation have only been established for dietary assessment of caloric intake and macro- and micronutrient intake, but not for TWI. There is only one question for plain water intake in the Harvard Willett Food Frequency Questionnaire, which only allows individuals to record a maximum of 1.5 L·d^−1^ with the allotted frequency options. This is not adequate considering the median water intake from liquids is 2.2 L for women and 3.0 L for men (4). Additionally, as mentioned previously, water consumption occurs throughout the day (10) and is often underreported on self-report tools that are not specific to beverages (11, 12). The eight occasions of consumption for plain water were included in our TWI-WFQ to accommodate individuals who drink more than 1.5 L·d^−1^ of plain water. These eight occasions also serve as a reminder for individuals to report water consumed throughout the day. Outside of plain water, all other beverages and foods were selected from the U.S. Department of Agriculture What We Eat in America Food Categories from NHANES 2009–2010 (23). Within each food category, some items included multiple foods with similar water content. For example, “mango, pineapple (1 cup)” was one item in the fruit category. The water content of 1 cup of mango and 1 cup of pineapple are 138 mL and 142 mL, respectively.

### Study Design

Participants visited the lab on nine separate occasions across 22 days with the second week serving as a wash-out period (Supplementary Table 1). A TWI-FQ was completed on day 1 to familiarize participants with the tool. Participants ingested D_2_O at the start of weeks one and three for determination of total body water and mean daily water turnover from the disappearance of D_2_O in the body water pool via the slope-intercept method (13, 18). The days following completion of weeks one and three (days 8, 22), participants completed the TWI-FQ for the previous seven days. Diet records (24) were completed daily during week two and analyzed to determine metabolic water (25, 26). Estimates of TWI from weeks one and three were compared between the D_2_O method and TWI-FQ method to assess the validity of the TWI-FQ. TWI estimates were compared between weeks one and three to assess reliability.

Baseline characteristics were collected on day one. Body mass was assessed with a scale, height was measured using a wall-mounted stadiometer, and body fat was measured via dual X-ray absorptiometry scan (Lunar Prodigy, GE Healthcare, Waukesha, WI).

### Total Water Intake: Frequency Questionnaire

**Figure 1:**
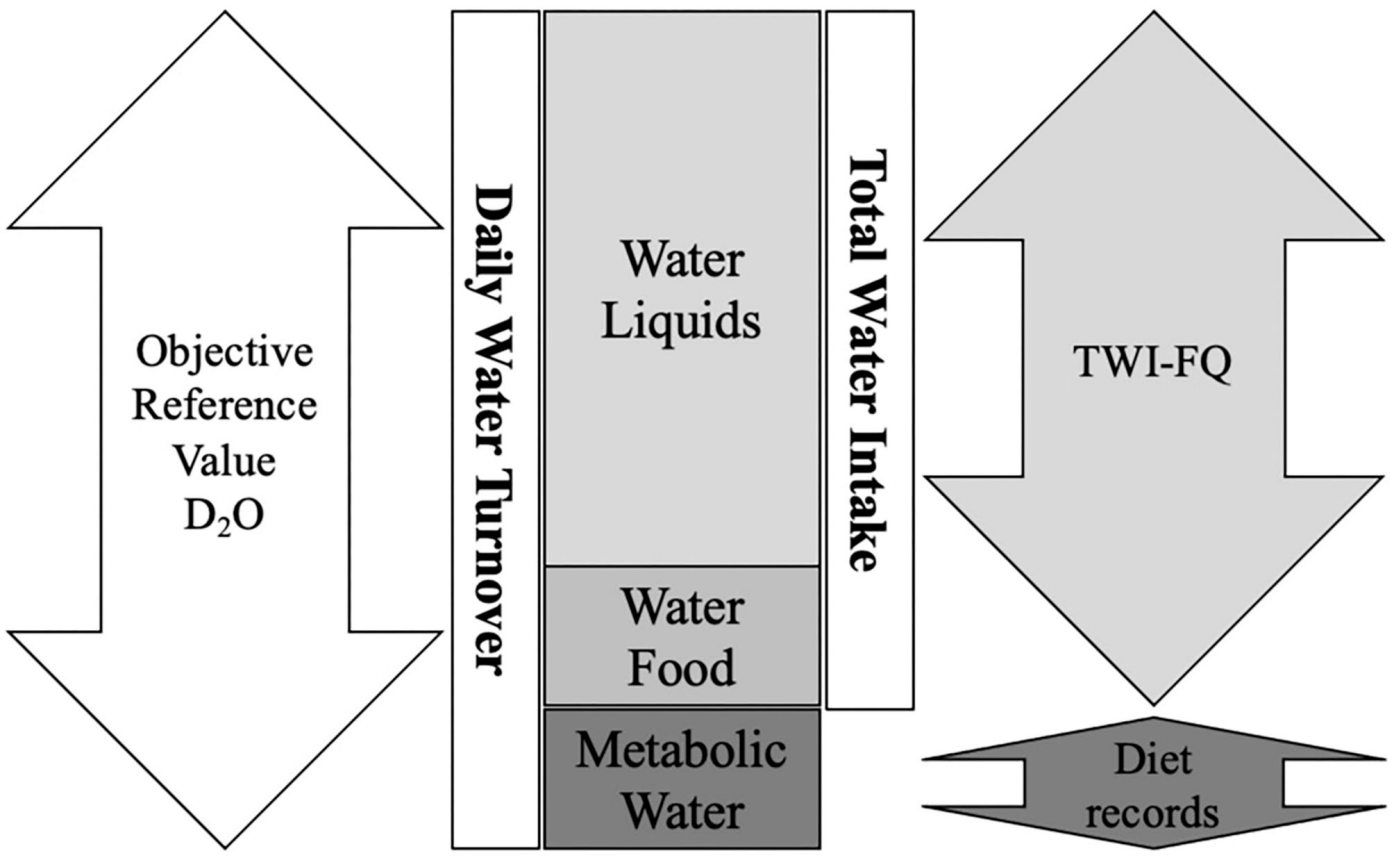
Estimation of total water intake using deuterium oxide dilution method and the total water intake frequency questionnaire. D_2_O, deuterium oxide; TWI-FQ, total water intake frequency questionnaire.

TWI-FQ were completed on days 1, 8, and 22. Day 1 served as a familiarization with the instrument, while participants retrospectively recalled water from liquids and foods for weeks 1 and 3 using TWI-FQs on days 8 and 22, respectively. The types and frequencies of liquids and foods consumed were entered into a customized spreadsheet and converted to mL (27). The volumes of liquids were converted to volumes of water based on standard water contents (e.g., 100 mL of milk = 89 mL water) (27). Reported volumes and frequencies were then used to determine mean daily water from liquids. Researchers converted reported quantities of foods to mL of water according to standard food water content and determined mean daily water from food based on calculated volumes and frequencies (Figure 1).

### Water Production From Metabolism

Participants recorded all food and liquid intake in 24-h diet records (24) every day of week 2 (days 8–14). For each item consumed, participants were instructed to record timing, portion size, method of preparation, number of servings, and any other pertinent information (i.e., brand name, restaurant, nutrient descriptors such as low-fat, condiments, etc.). Diet records were completed in real time, in contrast to diet recalls which can introduce error due to reliance on memory. Furthermore, multiple diet records were completed to increase the likelihood of capturing usual intake.

Diet records were analyzed with Nutrition Data System for Research software to determine the total energy intake and the proportions of energy that corresponded with each macronutrient. These values were then used to determine the volume of water generated through macronutrient oxidation using the following formula (25, 26):

$$\begin{array}{l} \text{Metabolic water }\left(\text{mL}\cdot {\text{d}}^{-\text{1}}\right)\text{=total energy expenditure}\\ \text{x }\left(\frac{\text{1}}{{\text{10}}^{\text{5}}}\right)\text{x }[\left(\%\text{fat x 0}.\text{119}\right)\text{+}\left(\%\text{protein x 0}.\text{103}\right)\\ \text{+}\left(\%\text{carbohydrate x 0}.\text{150}\right)\text{+}\left(\%\text{alcohol x 0}.\text{168}\right)] \end{array}$$

Total energy expenditure was assumed to be equivalent to total energy intake. Body weights measured on the first, second, and fifth days of both weeks were assessed to confirm weight stability and therefore confirm the aforementioned assumption was met.

### Total Water Intake: D_2_O Dilution

Participants provided a urine sample on day 1 immediately before D_2_O ingestion (0.1 g·kg^−1^ LBM, 99.9% deuterium, Cambridge Isotope Laboratories, Inc., Tewksbury, MA). The dose of D_2_O was added to a cup with 100 mL of water. Participants consumed the diluted tracer followed immediately by two additional 100 mL volumes of water ingested from the same cup to ensure tracer was consumed and not left on the cup. Participants returned on days 2 and 8 to provide additional urine samples. This process was repeated during week 3 on days 15, 16, and 22 with a D_2_O dose of 0.08 g·kg^−1^ LBM ingested at day 15 immediately after providing the urine sample. Samples were then analyzed via isotope ratio mass spectrometry (Micromass Isoprime DI, coupled with an Aquaprep system; Isoprime Ltd., Cheadle Hulme, UK) using the H_2_-water equilibration method to determine the ratio of deuterium to hydrogen (13, 28). The slope intercept method (29) was then used as previously described (13) to compute the volumes of total body water for weeks one and three from the dose ingested and the ratio of deuterium to hydrogen back-extrapolated at the time of ingestion, as well as water turnover from the disappearance of D_2_O from the body water pool. Finally, D_2_O TWI was calculated by subtracting metabolic water from water turnover.

### Sample Size Estimation

An a priori sample size of *n* = 75 was determined based on the desired accuracy of Bland-Altman limit of agreement estimates (30). Accuracy of estimates is determined by the standard error of 95% confidence intervals (CI) for the limits of agreement. Standard error (SE) was determined by $SE=\;\sqrt{}\left(3\;x\frac{SD^{2}}{n}\right)$, where SD is the standard deviation of the mean difference and n is the sample size. The 95*% CI* = ± 1.96 *x SE*. A sample size of 75 allows for 95*% CI* = ± 0.39 *x SD*.

### Statistical Analyses

Outcome variables were assessed for normality via Shapiro-Wilk test of normality, visual examination of the data (e.g., Q-Q plots, box plots, histograms), and skewness and kurtosis statistics. Non-normal data were analyzed non-parametrically. Analyses were conducted using commercial software (IBM SPSS Statistics Version 25.0.0). A jack-knife approach was employed using JMP Pro 15.2.0 (SAS Institute Inc.) to identify and examine the influence of outliers in the sample. Outliers were classified as mild (jack-knife distances > 2.5– ≤ 4.0) or severe (jack-knife distances > 4.0). A *P* <0.05 was considered statistically significant for all analyses. Data are presented as mean ± standard deviation.

Correlation and *t*-test analyses alone are not sufficient to assess validity between two measurement methods (31, 32). Therefore, we used paired *t*-tests to assess mean differences between measurements and a Bland-Altman plot to assess agreement between D_2_O and TWI-FQ to estimate daily fluid intake over weeks one and three. Bland-Altman analyses were conducted in accordance with methodology specific to multiple observations in which the true value of the primary outcome variable (i.e., TWI) is expected to vary over the observation period (33). This model accounts for mean difference (bias) between methods (TWI-FQ – D_2_O TWI) as well as variance in individual differences (between + within-subject variance). Individual differences between methods were plotted against the average of methods [(TWI-FQ – D_2_O TWI)/2], with repeated measurements treated as independent measurements (*n* = 196). Evaluation of the Bland-Altman plot within limits of agreement allowed us to understand the significance of bias of the TWI-FQ from the objective reference value D_2_O TWI. Kendall's tau was utilized to evaluate heteroscedasticity of the plot.

Reliability of the TWI-FQ to estimate TWI was assessed via related-samples Wilcoxon Signed Rank test, Spearman's correlation, and Intraclass correlation coefficient. Paired *t*-test and Spearman's correlation analyses were also conducted on D_2_O TWI to provide an indication of weekly variation in true TWI. To explore systematic bias in reliability, separate Bland-Altman plots (31) were created for D_2_O and TWI-FQ estimates of TWI. For each method, differences between repeated estimates of TWI (week 1 TWI – week 3 TWI) were plotted against the average of estimates from both weeks (*n* = 98).

## Results

Body mass was consistent within weeks (% of change in body mass: week 1, 0.05 ± 0.99%; week 2, 0.35 ± 1.19%; week 3, 0.11 ± 1.22%) with low coefficients of variance between the three measurements during all weeks (week 1, 0.54 ± 0.36%; week 2, 0.62 ± 0.41%; week 3, 0.60 ± 0.50%). Mean daily water turnover and the components that contribute to water turnover computed using data from D_2_O dilution and the TWI-FQ are presented in Table 2. Daily caloric intake during week 2 was 2,028 ± 523 kcal (range: 911–3,430 kcal).

**Table 2:** Mean daily water turnover and mean daily water intake by week and method.

	**Week 1**		**Week 2**	**Week 3**	
	**D_**2**_O**	**TWI-FQ**	**Food diaries**	**D_**2**_O**	**TWI-FQ**
Water turnover^a^, mL·d^−1^	3,680 ± 1,341			3,596 ± 1,275	
Metabolic water^a^, mL·d^−1^			264 ± 104		
Water from food^a^, mL·d^−1^		508 ± 258			490 ± 242
Water from liquids^a^, mL·d^−1^		2,624 ± 1,587			2,443 ± 1,358
**Total water intake^a^, mL·d**^**−1**^	**3,405** **±** **1,331^b^**	**3,132** **±** **1,665^c^**		**3,356** **±** **1,234^b^**	**2,933** **±** **1,425^c^**

*D_2_O, deuterium oxide dilution method; TWI-FQ, total water intake frequency questionnaire*.

a *Values are presented as mean ± standard deviation*.

b *Total Water Intake = Water Turnover – Metabolic Water*.

c *Total Water Intake = Water from Food + Water from Liquids*.

The jack-knife analysis identified eight mild outliers and three severe outliers across eight participants (63% male; age, 37 ± 13 y; BMI, 26.5 ± 5.9 kg·m^−2^) (Supplementary Table 2). All three severe outliers were found in males in week 1, while four mild cases were identified in each week. TWI was overestimated by the TWI-FQ in five of the eleven cases, two of which were identified as severe outliers. No outliers were excluded from validity or reliability analyses.

### Questionnaire Validity

**Figure 2:**
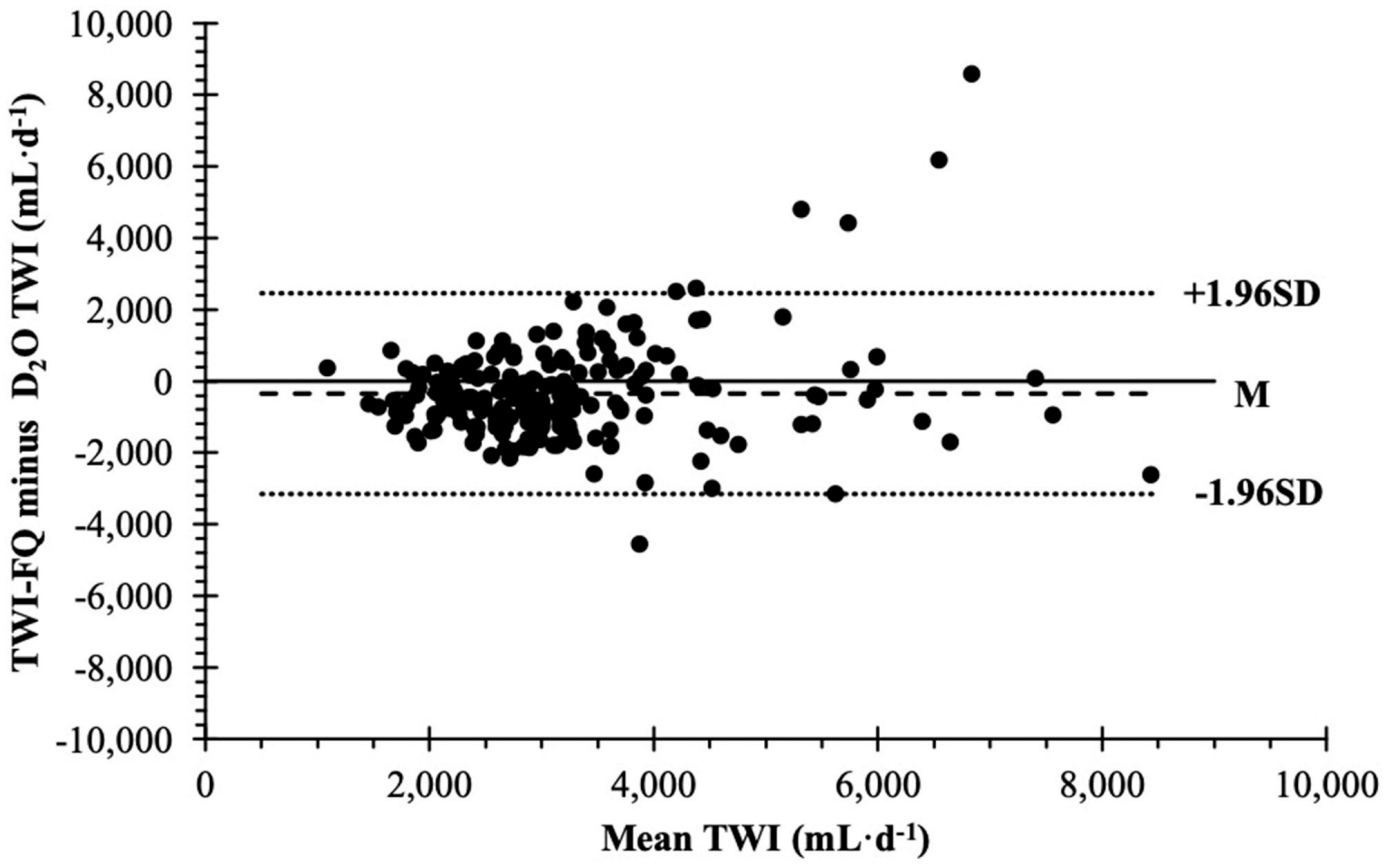
Bland-Altman plot of agreement between total water intake frequency questionnaire and deuterium oxide dilution method to estimate mean daily total water intake during two, one-week periods (*n* = 196). TWI, mean daily total water intake; TWI-FQ, total water intake frequency questionnaire; D_2_O, deuterium oxide dilution method; M, mean difference between methods (bias); SD, standard deviation of the mean difference.

TWI estimates were not different between methods during week 1 (*t*_[97]_ = 1.60, mean difference = −269 mL·d^−1^, 95% CI:−603, 65 mL·d^−1^, *P* = 0.1133), but were significantly different during week 3 (*t*_[97]_ = 3.71, mean difference = −431 mL·d^−1^, 95% CI: −661, −200 mL·d^−1^, *P* = 0.003). Combined TWI-FQ TWI estimates from both weeks significantly underestimated D_2_O estimates by −350 ± 1,431 mL·d^−1^ (95% CI: −551, −149 mL·d^−1^; Figure 2). Limits of agreement for the Bland-Altman plot were −3,155 and 2,455 mL·d^−1^. Kendall's tau was not significant (*r* = 0.076, *P* = 0.112), which indicates the data were not heteroscedastic.

### Questionnaire Reliability

**Figure 3:**
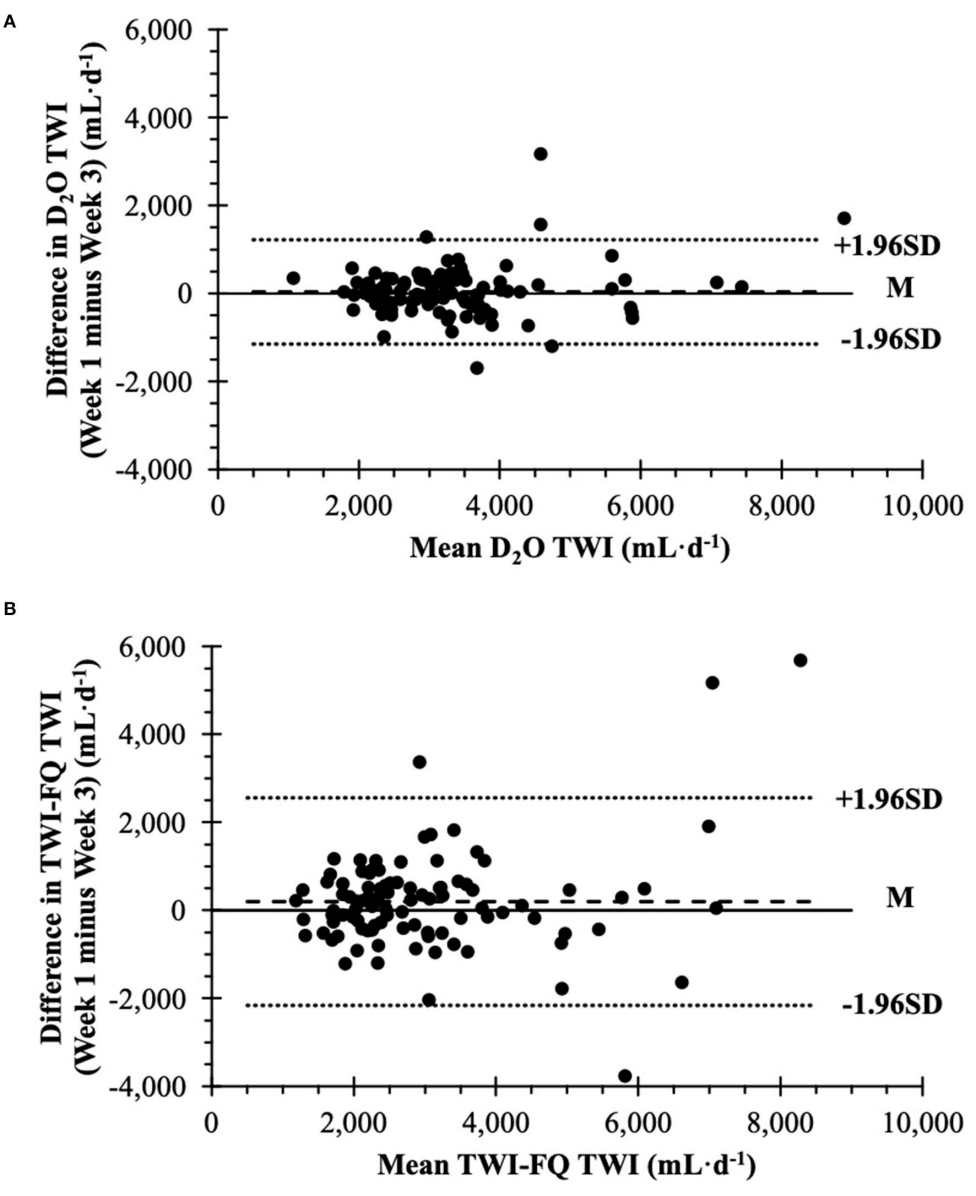
Bland-Altman plots of agreement between repeated estimates (week 1 and 3) of mean daily total water intake via **(A)** deuterium oxide dilution method and **(B)** total water intake frequency questionnaire (*n* = 196). TWI, mean daily total water intake; TWI-FQ, total water intake frequency questionnaire; D_2_O, deuterium oxide dilution method; M, mean difference between weeks (bias); SD, standard deviation of the mean difference.

D_2_O TWI was significantly correlated (r = 0.856, *P* <0.01) and was not different (*P* = 0.805) between weeks. Similarly, TWI-FQ TWI was significantly correlated (r = 0.707, *P* <0.01) and was not different (*P* = 0.115) between weeks 1 and 3. The Intraclass correlation coefficient for TWI-FQ was significant [ICC = 0.706, 95% CI: 0.591, 0.793; *F*_(97, 98)_ = 5.799, *P* <0.001], indicating moderate test-retest reliability. Based on Bland-Altman plots, the mean difference in D_2_O TWI estimates between weeks was 36 ± 593 mL·d^−1^ (95% CI: −83, 155 mL·d^−1^; Figure 3A). The mean difference in TWI-FQ TWI estimates between weeks was 198 ± 1,180 mL·d^−1^ (95% CI: −38, 435 mL·d^−1^; Figure 3B). Systematic bias in reliability was not observed for either method.

## Discussion

The purpose of this study was to assess the validity and reliability of a TWI-FQ to estimate TWI as compared to the objective reference value, D_2_O. The principle finding of this study is that the TWI-FQ consistently underestimated TWI. While this tool would not be suitable for individual assessment, the overall magnitude of bias may be acceptable for assessment at the sample-level. In this protocol, we utilized the isotopic tracer, D_2_O, as the rate of disappearance of D_2_O following enrichment is directly associated with water turnover and is not subject to homeostatic or inter-individual variations in metabolism (34). Accordingly, D_2_O is an unbiased measure of water turnover that is not subject to measurement error commonly seen in self-report data. Furthermore, we utilized Bland-Altman statistical analyses that accounted for variation between methods, between individuals, and between occasions (33). Most prior studies (14–17) have utilized correlation and *t*-test analyses, which are not independently sufficient to assess agreement between two methods for validity assessment (31, 32).

Due to the robustness of the D_2_O dilution method, we observed total body water as a percentage of body mass estimates within the ranges reported by the Institute of Medicine (43–73%, males 19–50 y; 41–60%, females 19–50 y) (4). Additionally, daily metabolic water production has been estimated to be ~250–350 mL·d^−1^ for sedentary individuals (4). Although metabolic water (264 ± 107 mL·d^−1^) was determined from diet records during the wash-out period in the current study, it still aligns with the aforementioned estimates. Confirmation of body weight stability for all participants across each week indicates that the assumption that TEE was equivalent to total energy intake was met for metabolic water calculations. Accordingly, we are confident the D_2_O TWI estimates reflect actual TWI.

The TWI-FQ significantly underestimated TWI by −350 ± 1,431 mL·d^−1^ compared to D_2_O. While the mean difference is clinically adequate, there is considerable variation in bias as evidenced by the standard deviation of 1,431 mL and limits of agreement allowing for underestimation of −3,155 mL·d^−1^ and overestimation up to 2,455 mL·d^−1^. The magnitude of these differences is substantial considering the Adequate Intake for water is 2.7 L·d^−1^ for women and 3.7 L·d^−1^ for men (4). Based on visual examination of Figure 3, these large differences appear to be driven, in part, by individuals who consume high amounts of TWI (≥4 L·d^−1^). In some cases, high amounts of TWI were accurately reported in the TWI-FQ (Supplementary Table 2). Large differences may in part be related to a learning curve as all three severe outliers were identified in the first week. Furthermore, most participants with outliers appeared to improve by week 3 (i.e., reduced from severe to mild outlier or no longer an outlier). Ultimately, outliers were a mixture of overestimation and underestimation with no clear association with subject characteristics (i.e., sex, age, BMI). Despite large variances, the TWI-FQ was still determined to be reliable due to moderate correlation between weeks (*r* = 0.725) and moderate test-retest reliability (ICC = 0.706). Systematic bias in TWI-FQ between weeks was not statistically significant as evaluated via Bland-Altman plot, in which the mean difference in TWI-FQ TWI estimates was 198 ± 1,180 mL·d^−1^.

The mean difference (36 ± 593 mL·d^−1^) between repeated D_2_O TWI estimates was minimal and non-significant. However, the acceptable limits of agreement (-1,149, 1,221 mL·d^−1^) are still large clinically and indicate a considerable degree of within-subject variance in week-to-week TWI. Additionally, mean D_2_O TWI was distributed across a wide range of volumes, between 1,000 and 9,000 mL·d^−1^, with the majority of mean D_2_O TWI falling between 1,000 and 4,500 mL·d^−1^. This indicates there is also a considerable degree of between-subject variance in D_2_O TWI, which was also captured by the TWI-FQ, as can be seen in Figures 3A,B. This magnitude of variance in TWI is not surprising as daily water needs can vary greatly between and within individuals depending on age, sex, diet, physical activity behaviors, climate, and culture (4). We purposefully recruited participants who were well-distributed across sex and age. Therefore, although the limits of agreement for the TWI-FQ validity assessment were large, these data indicate that the variance observed was compounded by within- and between-subjects' differences in water consumption habits.

Previous liquid questionnaires have been developed to assess fluid intake but not TWI (14–17, 19). While this TWI-FQ was designed specifically to assess water intake volume at population levels, previous questionnaires were developed primarily to assess energy intake from liquids (16, 17), grams or fluid ounces of individual and total liquids consumed (16, 17, 19), water intake and voiding habits for treatment of urinary tract symptoms (14), and water balance (15). Additionally, validation protocols for these questionnaires utilized imperfect reference instruments, such as 24-h diet records, which are subject to intake-related bias and correlated error (35). We used methods similar to the previous study to assess validity and reliability of the Liq.in^7^, which is a 7-day fluid record that required participants to record liquids and foods with high water content as they were consumed (18). Compared to D_2_O, the Liq.in^7^ underestimated water from liquids by −131 ± 845 mL·d^−1^. However, this assessment was based only on one week of data, the Bland Altman statistical analysis utilized did not account for within or between subject variation, and water from food was not included in this analysis. TWI was also assessed between the Liq.in^7^ and a 24-h dietary recall in Indonesian adolescents and adults using a Bland Altman analysis (12). An overestimation of 382 mL·d^−1^ was observed compared to the 24-h dietary recall with limits of agreement 1,600 and −2,300 mL·d^−1^. Although the limits of agreement were narrower than those in the current study, the difference was determined to be significant as 11% of values fell outside of these limits. The mean difference also increased with greater TWI, with underestimation of 139 mL·d^−1^ for the lowest quartile of TWI and overestimation of 1,265 mL·d^−1^ for the highest quartile of TWI. Thus, it appears individuals are less able to recall fluid intake accurately with greater consumption.

Our approach does not come without limitations. Metabolic water production was determined through self-reported data in 24-h diet records. Self-report dietary assessments are subject to error (e.g., difficulty interpreting handwriting, day-to-day variation in consumption, or misreporting of consumption) and can be burdensome to participants. However, metabolic water is a small component of water turnover (250–350 mL·d^−1^) (4) and over- or underestimation would not substantially impact the outcomes of this investigation. Furthermore, a prominent study in this field that determined water turnover in 458 adults (40–79 y) estimated metabolic water from the average macronutrient content of the diet based on a one-time 24-h recall in the general population in the US (26). In contrast, participants in the present investigation completed multiple 24-h diet recalls for metabolic water estimates.

The accuracy of the TWI-FQ may vary day-to-day, with TWI estimates that are more representative of days closer to the day of questionnaire completion. However, we were not able to evaluate this as participants are asked to recall consumption for the entire week rather than for each day of the week. Similarly, the D_2_O method utilizes three urine samples to determine an average daily TWI for the 7-day period and does not allow for estimation for each specific day. Furthermore, we were not able to evaluate potential differences in validity or reliability of the TWI-FQ by age or sex as this study was not powered for these comparisons. Finally, we were not able to validate whether the TWI-FQ is sensitive to change in TWI. Therefore, this tool may not be suitable for use in intervention studies designed to change TWI, particularly if detection of small changes is desired.

In conclusion, the TWI-FQ may be a useful tool to assess population-level TWI behaviors. Due to the large variances observed, the TWI-FQ should not be utilized to assess individual-level TWI behaviors in which greater accuracy may be needed. Utilization of the TWI-FQ to assess population-level TWI may allow investigators to better determine relationships between liquid intake, hydration, and health. Moreover, the TWI-FQ could be utilized in conjunction with multiple 24-h diet recalls/records to better reflect water from food and subsequently TWI. Several studies have successfully improved accuracy of self-report dietary data through combining 24-h diet recall/records with food frequency questionnaires (36). The findings of this study can only be generalized to individuals 19–65 y. Further investigation is needed to assess application of the TWI-FQ in different geographical regions, climates, cultures, activity levels, and age groups.

## Data Availability Statement

The raw data supporting the conclusions of this article will be made available by the authors, without undue reservation.

## Ethics Statement

The studies involving human participants were reviewed and approved by University of Arkansas, IRB. The patients/participants provided their written informed consent to participate in this study.

## Author Contributions

The authors' responsibilities were as follows–SK, FP, IG, and EP designed research (project conception, development of overall research plan, and study oversight). EJ, LJ, CC-J, and JA conducted research (hands-on conduct of the experiments and data collection). AC, EJ, FP, SK, and AM analyzed data or performed statistical analysis. AC and SK wrote paper. SK had primary responsibility for final content. All authors have read and approved the final manuscript.

## Conflict of Interest

EJ had received grants from Danone Research and FP had served as member of the scientific advisory board of Danone Research. IG and EP were Danone Research employees. SK had served as scientific consultant for Quest Diagnostics and Standard Process and had served as a member of the scientific advisory board of Danone Research, and had active grants with Danone Research and Standard Process. The remaining authors declare that the research was conducted in the absence of any commercial or financial relationships that could be construed as a potential conflict of interest.
