# Supplementary figures and images for: Validity and Reliability of a Water Frequency Questionnaire to Estimate Daily Total Water Intake in Adults
Source: Front Nutr. 2021 Jun 14;8:676697. doi: 10.3389/fnut.2021.676697 (PMC8236537; doi:10.3389/fnut.2021.676697)

## Supplementary Material

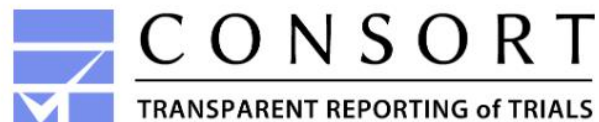

### CONSORT 2010 Flow Diagram

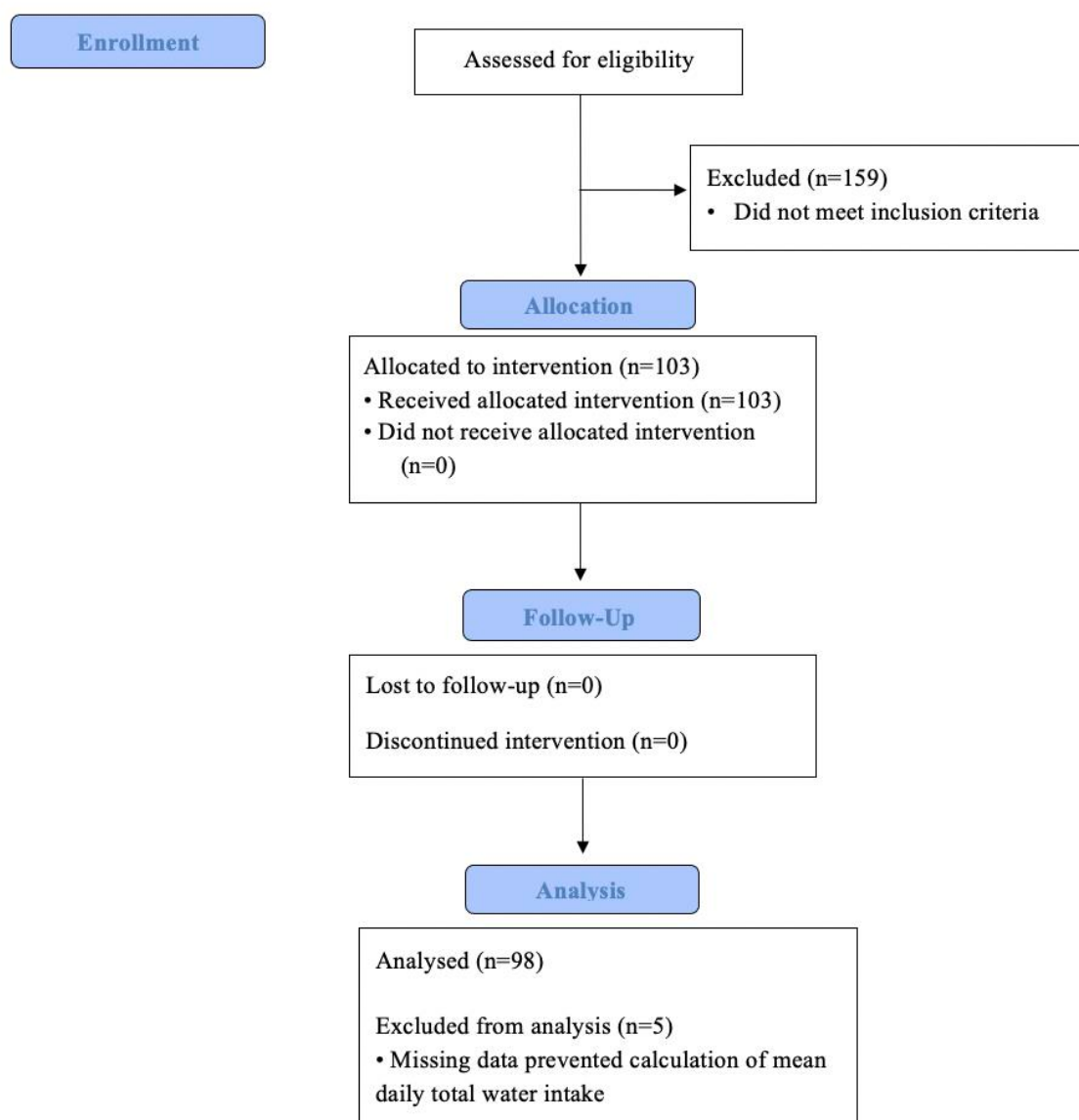

**Supplementary Figure 1.** Study CONSORT Diagram.

Supplement: Supplementary file 1 [file Data_Sheet_1.pdf]
