# Supplementary material for: Validity and Reliability of a Water Frequency Questionnaire to Estimate Daily Total Water Intake in Adults
Source: Front Nutr. 2021 Jun 14;8:676697. doi: 10.3389/fnut.2021.676697 (PMC8236537; doi:10.3389/fnut.2021.676697)
Supplement: Supplementary file 2 [file Table_1.DOCX]

| **Supplementary Table 1.** Study protocol. | | | | | | | | | | | | | | | | | | | | | | | | | | |
| --- | --- | --- | --- | --- | --- | --- | --- | --- | --- | --- | --- | --- | --- | --- | --- | --- | --- | --- | --- | --- | --- | --- | --- | --- | --- | --- |
|  | **WEEK 1** | | | | | | |  | | **WEEK 2** | | | | | | |  | **WEEK 3** | | | | | | |  | **WEEK 4** |
| Day | **1** | **2** | **3** | **4** | **5** | **6** | **7** |  | | **8** | **9** | **10** | **11** | **12** | **13** | **14** |  | **15** | **16** | **17** | **18** | **19** | **20** | **21** |  | **22** |
| Lab visit | **V1** | **V2** |  |  | **V3** |  |  |  | | **V4** | **V5** |  |  |  |  |  |  | **V6** | **V7** |  |  | **V8** |  |  |  | **V9** |
| Screening | x |  |  |  |  |  |  | |  |  |  |  |  |  |  |  |  |  |  |  |  |  |  |  |  |  |
| Drink D_2_O | D1 |  |  |  |  |  |  | |  |  |  |  |  |  |  |  |  | D2 |  |  |  |  |  |  |  |  |
| Weight | x | x |  |  | x |  |  | |  | x | x |  |  |  |  |  |  | x | x |  |  | x |  |  |  | x |
| Morning urine | x | x |  |  |  |  |  |  | |  |  |  |  |  |  |  |  | x | x |  |  |  |  |  |  | x |
| 24-h diet record |  |  |  |  |  |  |  |  | | x | x | x | x | x | x | x |  |  |  |  |  |  |  |  |  |  |
| TWI-FQ | x |  |  |  |  |  |  |  | | x |  |  |  |  |  |  |  |  |  |  |  |  |  |  |  | x |
| Abbreviations: D1, D_2_O dose 0.10 g/kg lean body mass 99.9% deuterium; D2, D_2_O dose 0.08 g/kg lean body mass, 99.9% deuterium; D_2_O, deuterium oxide. | | | | | | | | | | | | | | | | | | | | | | | | | | |
