# Supplementary material for: Validity and Reliability of a Water Frequency Questionnaire to Estimate Daily Total Water Intake in Adults
Source: Front Nutr. 2021 Jun 14;8:676697. doi: 10.3389/fnut.2021.676697 (PMC8236537; doi:10.3389/fnut.2021.676697)
Supplement: Supplementary file 3 [file Table_2.DOCX]

| **Supplementary Table 2**. Selected demographics and total water intake for participants with jack-knife outliers. | | | | | | | | | | | |
| --- | --- | --- | --- | --- | --- | --- | --- | --- | --- | --- | --- |
|  | **Demographics** | | |  | **Week 1** | | |  | **Week 3** | | |
| **Participant** | **Sex** | **Age, y** | **BMI, kg·m^2^** |  | **D_2_O, mL** |  | **TWI-FQ, mL** |  | **D_2_O, mL** |  | **TWI-FQ, mL** |
| 1^a^ | Male | 23 | 39.6 |  | 2,550 |  | 11,127 |  | 2,956 |  | 5,449 |
| 2^b^ | Male | 28 | 22.6 |  | 6,162 |  | 1,594 |  | 3,001 |  | 2,511 |
| 3^c^ | Female | 24 | 23.4 |  | 7,208 |  | 4,041 |  | 6,967 |  | 5,824 |
| 4^a^ | Male | 31 | 20.6 |  | 3,469 |  | 9,629 |  | 3,776 |  | 4,462 |
| 5^d^ | Male | 52 | 27.4 |  | 9,746 |  | 7,128 |  | 8,042 |  | 7,081 |
| 6^c^ | Female | 30 | 28.1 |  | 7,504 |  | 5,789 |  | 7,368 |  | 7,440 |
| 7^b^ | Male | 45 | 27.1 |  | 3,537 |  | 7,940 |  | 4,260 |  | 6,046 |
| 8^e^ | Female | 58 | 23.2 |  | 2,862 |  | 3,929 |  | 2,921 |  | 7,709 |
| Abbreviations: BMI, body mass index; D2O, deuterium oxide dilution method; TWI-FQ, total water frequency questionnaire. Mild outlier, jack-knife distance > 2.5 – ≥ 4.0; severe outlier, jack-knife distance > 4.0  ^a^Severe outlier week 1 only  ^b^Mild outlier week 1 only  ^c^Mild outlier weeks 1 and 3  ^d^Severe outlier week 1, mild outlier week 3  ^e^Mild outlier week 3 only | | | | | | | | | | | |
|  |  |  |  |  |  |  |  |  |  |  |  |
|  |  |  |  |  |  |  |  |  |  |  |  |
|  |  |  |  |  |  |  |  |  |  |  |  |
